# Performance of health laboratories in provision of HIV diagnostic and supportive services in selected districts of Tanzania

**DOI:** 10.1186/s12913-017-2030-9

**Published:** 2017-01-23

**Authors:** Deus S. Ishengoma, Mathias L. Kamugisha, Acleus S. M. Rutta, Gibson B. Kagaruki, Andrew M. Kilale, Amos Kahwa, Erasmus Kamugisha, Vito Baraka, Celine I. Mandara, Godlisten S. Materu, Julius J. Massaga, Stephen M. Magesa, Martha M. Lemnge, Leonard E. G. Mboera

**Affiliations:** 1 National Institute for Medical Research, Tanga Research Centre, P. O. Box 5004, Tanga, Tanzania; 2 National Institute for Medical Research, Tukuyu Research Centre, P. O. Box 538, Tukuyu, Tanzania; 3 National Institute for Medical Research, Muhimbili Research Centre, P. O. Box 3436, Dar es Salaam, Tanzania; 4 Catholic University of Health and Allied Sciences, P. O. Box 1464, Mwanza, Tanzania; 5 National Institute for Medical Research, P. O. Box 9653, Dar es Salaam, Tanzania; 6 National Institute for Medical Research, Amani Research Centre, P. O. Box 81, Muheza, Tanzania

## Abstract

**Background:**

Roll-out and implementation of antiretroviral therapy (ART) necessitated many countries in Sub-Saharan Africa to strengthen their national health laboratory systems (NHLSs) to provide high quality HIV diagnostic and supportive services. This study was conducted to assess the performance of health laboratories in provision of HIV diagnostic and supportive services in eight districts (from four regions of Iringa, Mtwara, Tabora and Tanga), after nine years of implementation of HIV/AIDS care and treatment plan in Tanzania.

**Methods:**

In this cross-sectional study, checklists and observations were utilized to collect information from health facilities (HFs) with care and treatment centres (CTCs) for HIV/AIDS patients; on availability of laboratories, CTCs, laboratory personnel, equipment and reagents. A checklist was also used to collect information on implementation of quality assurance (QA) systems at all levels of the NHLS in the study areas.

**Results:**

The four regions had 354 HFs (13 hospitals, 41 Health Centres (HCs) and 300 dispensaries); whereby all hospitals had laboratories and 11 had CTCs while 97.5 and 61.0% of HCs had both laboratories and CTCs, respectively. Of the dispensaries, 36.0 and 15.0% had laboratories and CTCs (mainly in urban areas). Thirty nine HFs (12 hospitals, 21 HCs and six dispensaries) were assessed and 56.4% were located in urban areas. The assessed HFs had 199 laboratory staff of different cadres (laboratory assistants = 35.7%; technicians =32.7%; attendants = 22.6%; and others = 9.1%); with >61% of the staff and 72.3% of the technicians working in urban areas. All laboratories were using rapid diagnostic tests for HIV testing. Over 74% of the laboratories were performing internal quality control and 51.4% were participating in external QA programmes. Regional and district laboratories had all key equipment and harmonization was maintained for Fluorescence-Activated Cell Sorting (FACS) machines. Most of the biochemical (58.0%) and haematological analysers (74.1%) were available in urban areas. Although >81% of the equipment were functional with no mechanical faulty, 62.6% had not been serviced in the past three years.

**Conclusion:**

Diagnostic and supportive services for HIV were available in most of the HCs and hospitals while few dispensaries were providing the services. Due to limitations such as shortage of staff, serving of equipment and participation in QA programmes, the NHLS should be strengthened to ensure adequate human resource, implementation of QA and sustainable preventive maintenance services of equipment.

## Background

HIV/AIDS is still a leading cause of morbidity and mortality among adult individuals globally and causes more than 1.1 million deaths mainly among individuals aged ≥15 year [1, 2]. Although a significantly large amount of resources has been invested in HIV diagnostics and therapeutics over the past decade, more than 2.1 million newly acquired infections were reported in 2015, despite a declining trend globally [1]. In Tanzania, HIV remains the main public health problem with enormous impact on human health and subsequently on economic development [3]. Efforts focusing on prevention and treatment are still required to contain the pandemic and support people living with HIV/AIDS (PLHA). From October 2004, Tanzania launched an HIV care and treatment plan which had a target of putting more than 400,000 AIDS patients on antiretroviral therapy (ART) by the end of 2008 [4]. In Tanzania, the HIV care and treatment plan required a major improvement of the health system including health laboratories for not only providing ARTs, but also monitoring the disease in 1.2 million people infected with HIV [4].

Strong and efficient national health laboratory systems (NHLSs) and networks capable of providing high quality services are critical components of the health system of any country. The NHLS are not only important for routine diagnoses, care, treatment and surveillance of HIV/AIDS but also for proper management and surveillance of other diseases. However, the NHLSs of most resource-poor countries (RPCs) are faced with multiple challenges including shortage of financial resources, skilled and trained personnel, inadequate infrastructure (including reliable electricity, water supply and physical infrastructure) and inadequate laboratory equipment [5–8]. The NHLSs also suffer from poor supply chain management for consumables and reagents, poor system for equipment maintenance, lack of clear laboratory policies and insufficient laboratory management skills [9–11]. Furthermore, the NHLSs of most RPCs have weak quality management systems and accreditation of laboratories, laboratory information systems and bio-safety and waste management programmes [12, 13]. Similarly in Tanzania, the NHLS has for a long time suffered from many problems which made it incapable of providing basic services to address the major diseases including HIV/AIDS, tuberculosis and malaria [14, 15].

With increased funding in recent years, strengthening of the NHLSs has been prioritized as part of the efforts to deal with HIV/AIDS pandemic. Before and after the roll-out of ART, RPCs embarked on laboratory capacity improvement and strengthening programme to support provision of antiretroviral drugs (ARVs), monitor patients’ progression, surveillance of the disease and detection of potential development of drug resistance [11, 15–17]. Tanzania also embarked on laboratory capacity strengthening, following an assessment conducted in 2002 which showed that the NHLS needed upgrading in general infrastructure, personnel, equipment, and laboratory management and quality assurance systems [18, 19]. Furthermore, standardization of different processes involved in HIV diagnosis was undertaken to ensure that overall management, equipment maintenance, human resources, quality management, training, data management, sample transport and monitoring and evaluation were properly undertaken and coordinated [18]. The national guidelines for voluntary counselling and testing were developed in 2004 to provide guidance on HIV diagnosis, quality control (QC) and quality assurance (QA) procedures to be done by health facilities of different levels [20]. In addition, the national HIV reference laboratory and Zonal centres were established for conducting external quality assurance (EQA) of HIV diagnostic services and supporting different laboratories in the country [19, 21].

Despite substantial investments and capacity building of the NHLSs in Tanzania, there has been limited information on the performance of the laboratories, particularly availability, accessibility and quality of services for HIV diagnosis in the country. Studies conducted in different regions after the roll out of care and treatment plan for HIV/AIDS in Tanzania, showed that most of the laboratories were not properly adhering to good laboratory practice (GLP), with poor laboratory quality control for HIV testing reagents, and were not performing internal and external quality control [22, 23]. Other studies conducted in similar settings also showed that health personnel were dissatisfied with HIV diagnostic services provided by their laboratories with higher dissatisfaction rates in public health facilities than in the private sector [24]. Due to differences in performance of HIV diagnostic methods, different HIV testing algorithms are usually recommended [25, 26], indicating that frequent assessment of HIV diagnostic tests is needed to ensure compliance with the national and international guidelines at all levels of the health care system. The study reported in this paper was therefore conducted in areas with varying levels of HIV transmission to assess the performance of laboratories in the provision of HIV diagnostic and supportive services after implementation of HIV/AIDS care and treatment plan for nine years in Tanzania. The findings of this study are envisaged to provide useful evidence for addressing policy challenges for strengthening the availability, accessibility and quality of HIV diagnostic and related supportive services in Tanzania.

## Methods

### Study area

This study was conducted between September and December 2013 in four regions of Iringa, Mtwara, Tabora and Tanga in Mainland Tanzania and it involved eight districts, two in each region. The eight districts included Iringa Rural and Iringa Urban (in Iringa region); Masasi and Mtwara (Mtwara); Igunga and Tabora (Tabora); and Muheza and Tanga (Tanga region). Study health facilities (HFs) with HIV/AIDS laboratory services were selected from the districts and they were located in rural and urban/peri-urban areas, and both public and private facilities.

It was assumed that general health and laboratory services would differ among regions in Tanzania due to differences in the burden of HIV and other diseases; and also as previously shown that the services differ with geographical location resulting in disproportionately poor laboratory services in rural areas [27, 28]. Therefore, the study targeted areas with varying HIV transmission and districts located in both urban and rural areas in Mainland Tanzania. The regions were selected by stratified random sampling whereby three groups were formed to include regions with low, moderate and high HIV transmission based on the 2011/2012 Tanzania HIV and Malaria Indicator Survey (THMIS) [3]. According to the 2011/2012 Survey [3], Njombe region had the highest HIV prevalence (14.8%) in Mainland Tanzania while Manyara had the lowest (1.5%) and the national average was 5.1%. Four regions were randomly selected for the study and they included one region (Iringa, with prevalence of 9.1%) among the four regions with high prevalence of HIV (7.4–14.8%) and two regions of Tabora (5.1%) and Mtwara (4.1%) among the 15 regions with moderate HIV prevalence (3.4–7.0%). Tanga (2.4%) was selected to represent the six regions with low prevalence of HIV (1.5–3.3%). In each region, a purposive sampling was utilized to select two districts from urban and rural areas while convenient sampling was used to select the study health facilities due to low number of dispensaries with care and treatment centres (CTCs) for HIV/AIDS. A maximum of six health facilities with CTCs and/or the capacity for laboratory diagnostic services for HIV were targeted in each district to cover different levels of laboratories including public and private laboratories, and those located within dispensaries, health centres and hospitals.

### Study design and data collection

In this cross-sectional study, checklists and observations were utilized for data collection. Total quality management approach covering quality control, quality assurance and quality improvement strategies [14, 29] was utilised for development of techniques for sampling and data collection. Modifications of study tools were done to include variables which are commonly evaluated in the new laboratory accreditation scheme in the African Region under the World Health Organisation (WHO) [9]. Key components of quality assurance and quality improvement systems aimed at assessing the facilities/infrastructure, equipment, reagents, laboratory management, personnel, standard operating procedures (SOPs) and laboratory guidelines, and quality assurance programs.

Two types of checklists were used for data collection at each HF whereby the assessment was done by experienced laboratory scientists/technologists. The information was obtained by observation, review of records or from the head of the laboratory. The first checklist covered personnel, availability of equipment and reagents and general infrastructure. This tool helped to collect information on the number of laboratory staff, their qualifications and employment conditions (full or part time). It was also used to obtain information on the different types and number of equipment available in each laboratory, their maintenance and working status. Any equipment was categorised as functional if it had no major mechanical/functional fault, and thus could be used for analysis of specimen at the time of the visit. Other information collected included availability and stock levels of diagnostic reagents, HIV test kits, participation in EQA programmes, laboratory premises and infrastructure and information on any new diagnostic techniques adopted within five years before the study.

Furthermore, data on SOPs and general / quality control (QC) guidelines, as well as their implementation was assessed. Further assessment covered availability and application of guidelines for waste management, the national structure of laboratory management and other key guidelines for HIV diagnostic and supportive services. The second checklist was used to assess and obtain information on equipment maintenance, covering issues such as cleanliness, storage after use, availability of maintenance schedule, records of service done, functional status, usability and availability of essential replacement components.

### Data management and analysis

Data collected was double entered into a Microsoft Access database which incorporated in-built consistence checks and validation features. Manual cleaning of the data was performed before analysis using STATA version 12 (STATA Corp Inc., TX, USA). Summary statistics were generated to show frequencies of key variables by region, district and location of health facility (e.g. urban vs. rural). Comparison was also made for HFs located in regions with high, medium and low HIV transmission and at facilities of different levels (regional and district/other hospitals and health centres/dispensaries). Continuous variables such as number of laboratory personnel in the different groups of HFs (e.g. those located in urban vs. rural or different HIV transmission) was compared using non-parametric tests (Kruskal-Wallis or Wilcoxon rank sum test) and reported as median with their corresponding inter-quartile range (IQR). Categorical data were compared using Chi-square test and findings were presented in tables and figures. *P*-value ≤0.05 was considered to be significant.

### Ethical considerations

Ethical approval was sought and obtained from the Medical Research Coordination Committee (MRCC) of the National Institute for Medical Research (NIMR). Permission to conduct the study in the four regions was sought from relevant authorities including the offices of the regional and district medical officers while permission to collect data from the HFs was obtained from the heads of the respective facilities. For each participant interviewed (head of HF/laboratory), both oral and written informed consent were provided before the interview.

## Results

### Health facilities, laboratories and staff

**Fig. 1:**
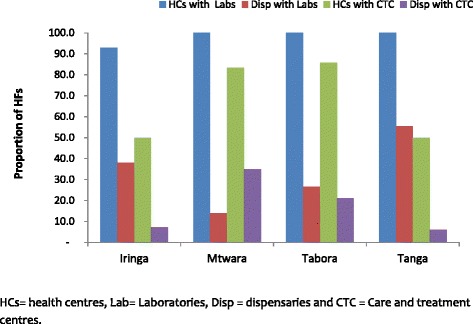
Availability of laboratories and care and treatment centres for HIV/AIDS at health centres and dispensaries in the study regions

The four regions had a total of 354 health facilities (HFs) which included 13 (3.7%) hospitals, 41 (11.6%) health centres (HCs) and 300 (84.7%) dispensaries; and the majority (60.2%) were located in rural areas (Table 1). Whereas all 13 hospitals and 40 (97.6%) HCs (except one in Iringa rural district) had laboratories, only 108 (36.0%) dispensaries had laboratory facilities. A significantly large proportion of dispensaries located in rural areas (86.6%) had no laboratories while only 27.2% of the dispensaries in the urban areas had no laboratories (*p* < 0.001). Care and treatment centres (CTCs) for people living with HIV/AIDS (PLHA) were available in 81 (22.9%) HFs including 11 (84.6%) hospitals (two hospitals in Iringa had no CTCs), 25 (61.0%) HCs and only 45 (15.0%) dispensaries. Although there was no significant difference in the number of HCs with CTCs among those located in urban *vs.* rural areas (*p* = 0.656), a significantly large number of the dispensaries without CTCs were located in rural (90.9%) compared to urban areas (75.5%) (*p* = 0.001) (see Table 1). All HCs in Tanga and 92.9% in Iringa had laboratories but only 50% of the HCs in these regions had CTCs; while the majority of HCs in both Mtwara (83.3%) and Tabora (85.7%) had CTCs. Furthermore, the majority of the dispensaries with CTCs (>21%) were in Mtwara and Tabora; regions with moderate HIV transmission (Fig. 1). A total of 39 health facilities were selected for this study and they included 12 hospitals, 21 health centres, and six dispensaries; and 56.4% of these were located in urban areas (Table 2). The selected facilities accounted for 48.1% of the health facilities with CTCs (*n* = 81).

**Table 1:** Number of health facilities in the four regions and availability of laboratories and care and treatment centres for patients with HIV/AIDS

District	Hospitals			Health Centres			Dispensary		
	N	With Labs	With CTC	N	With Labs	With CTC	N	With Labs	With CTC
Rural									
Iringa Rural (*n* = 72)	1	1	1	10	9	5	61	9	0
Masasi (*n* = 32)	2	2	2	3	3	3	27	3	3
Igunga (*n* = 62)	2	2	2	5	5	4	55	4	9
Muheza (*n* = 47)	1	1	1	3	3	2	43	9	5
Sub-Total (*n* = 213)	6	6	6	21	20	14	186	25	17
Urban									
Iringa Urban (*n* = 30)	3	3	1	4	4	2	23	23	6
Mtwara (*n* = 20)	1	1	1	3	3	2	16	3	12
Tabora (*n* = 39)	2	2	2	2	2	2	35	20	10
Tanga (*n* = 52)	1	1	1	11	11	5	40	37	0
Sub-Total (*n* = 141)	7	7	5	20	20	11	114	83	28
Overall Total (*n* = 354)	13	13^a^	11^b^	41	40^a^	25^b^	300	108^a^	45^b^

*N* = Total number of health facilities, *Labs* Laboratories, *CTC* Care and treatment Centre, *n* number of health facilities in each district and in rural/urban areas. Overall, 161 (45.5%)^a^ health facilities had laboratories and only 81 (24.9%)^b^ had CTCs; 76.1 and 82.6% of the HFs located in rural areas had no laboratories and CTCs, respectively

**Table 2:** Distribution of the 39 study heath facilities by ownership and location

Item	Iringa, n (%)	Mtwara, n (%)	Tabora, n (%)	Tanga, n (%)	Total, n (%)
Level of HF					
Dispensary	2 (20.0)	3 (33.3)	0 (0)	1 (10.0)	6 (15.4)
Health Centre	4 (40.0)	3 (33.3)	7 (70.0)	7 (70.0)	21 (53.8)
Hospital	4 (40.0)	3 (33.3)	3 (30.0)	2 (20.0)	12 (30.8)
Ownership					
Public	5 (50.0)	6 (66.7)	6 (60.0)	8 (80.0)	25 (64.1)
Private	5 (50.0)	3 (33.3)	4 (40.0)	2 (20.0)	14 (35.9)
Location					
Rural	4 (40.0)	4 (44.0)	5 (50.0)	4 (40.0)	17 (43.6)
Urban	6 (60.0)	5 (56.0)	5 (50.0)	6 (60.0)	22 (56.4)

*n* = number of health facilities, *%* percentage, *HF* Health facilities

The assessed facilities had 199 laboratory staff whereby the median number of staff was 3 (inter-quartile range, IQR = 2 - 6), with the highest number of staff at regional laboratories. The median number of laboratory staff was higher in Tanga (median = 4, IQR = 2–7) and Tabora (median = 4, IQR = 3-7) while the two regions of Iringa (median = 3, IQR = 2 – 7) and Mtwara (median = 3, IQR = 1 – 12) had fewer number of laboratory personnel. The differences in the number of staff working in laboratories located in areas with different HIV transmission was not statistically significant (*p* > 0.893). However, the number of staff was significantly higher in laboratories located in urban areas (median = 4, IQR = 3 – 6) compared to those located in rural areas (median = 2, IQR = 1 – 9), (*p* = 0.016).

**Fig. 2:**
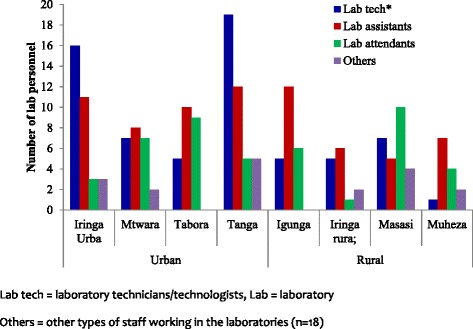
Number of laboratory personnel available at the study health facilities in the four regions

With the exception of one regional laboratory which had only five personnel, the rest had ≥12 laboratory staff (maximum 18). The laboratories located at district/other hospitals had ≥9 people (ranged from 9 to 12) while two laboratories in private hospitals had fewer staff with three and eight people each. The 27 primary laboratories (HCs and dispensaries) had lower number of staff with one person in six HFs (including 5 HCs and one dispensary), two in five, three in six and four people in nine facilities. In two of the HCs with one laboratory staff, the available person was a laboratory attendant. Three facilities [(7.7%); two HCs and one dispensary] had no qualified laboratory staff and the available personnel were either laboratory auxiliary staff or nurses/nurse assistant. Laboratory assistants accounted for 35.7% of the laboratory staff followed by technologists/technicians (32.7%) and laboratory attendants (22.6%). Other types of staff (9.1%, *n* = 18) included laboratory auxiliary staff (4), nurses (3), medical attendants (2), laboratory scientists (3), data entry clerks (3) and volunteers (3). Most of the laboratory staff (61.3%) and technologists/technicians (72.3%) were working in urban areas (Fig. 2).

### Availability and type of services offered by the health facilities

**Fig. 3:**
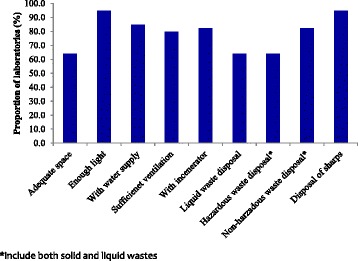
Physical infrastructure of the laboratories and waste disposal facilities

Despite the availability of CTCs, three laboratories including a district hospital in Iringa were not performing HIV diagnosis at the time of the visit. The remaining laboratories (92.3%, *n* = 36) were using rapid diagnostic tests (Determine and Uni-Gold test kits) for HIV testing. Only three laboratories (7.7%) adopted at least one new diagnostic technique in the past five years and these included ELISA for HIV diagnosis in infants, collection of dried blood spots for HIV diagnosis among infants and implementation of counselling and testing for HIV. Twenty nine laboratories (74.4%) reported that they were performing internal quality control and they included all regional laboratories, seven district/other hospitals’ laboratories (one was not doing QC) and the rest were low level laboratories (HCs and dispensaries). Whereas most of the laboratories mentioned that they were using known control samples or testing a new batch of kits, three laboratories (one hospital and two HCs) reported that they were doing QC of CD4, and biochemistry and haematology reagents only. Only 22 laboratories (56.4%) including all regional and three district hospitals reported that they were participating in external quality assurance programmes. Although majority of the laboratories had sufficient physical infrastructure for provision of laboratory services, about 36% of the laboratories had insufficient facilities for waste disposal and limited laboratory space (Fig. 3).

### Availability and functional status of equipment

Equipment available in the laboratories included biochemical analyzers (31), Fluorescence-Activated Cell Sorting (FACS) machines (14), haematology analyzers (27), HaemoCue® machines (28), microscopes (16), Pima® CD4 machines (11), refrigerators (30) and others (31). All regional and district/other hospitals’ laboratories had at least one biochemistry analyser while only 11 HCs (52.4%) and two dispensaries from Iringa urban and Mtwara urban districts had a biochemistry analyser. Each of the four regional laboratories had at least one haematology analyser (with two in Tanga and Mtwara) and the four districts/other hospitals had one each, with fewer analysers at primary level laboratories (Table 3). With the exception of one regional laboratory which had only one FACS machine, the rest had two and the machines were similar (FACS Calibre and FACS Counter). All district laboratories and two other laboratories located at hospitals had FACS counters (one in each of the facilities) and only one health Centre from Tanga had a FACS counter (Table 3). Different brands of biochemical and haematology analyzers were found in the laboratories while all Pima® CD4 machines were similar. Over 81% of the equipment was functional with no mechanical fault. However, most of the equipment (62.6%) including one FACS machine, 12 (38.7%) biochemistry and 11 (40.7%) haematology analysers had not been serviced in the past three years. Four FACS machines (28.6%) including two in Iringa and one in each of the two districts of Mtwara urban and Tabora were not functional at the time of the study. Three out of the 11 (27.3%) Pima® CD Machines were not functional. Of the biochemical analysers, 18 (58%) were owned by health facilities located in urban areas and 7 (22.6%) were not functional. Similarly, 20 (74.1%) haematology analysers were found in laboratories located in urban areas and 6 (22.2%) were not functional.

**Table 3:** Type of equipment available at the 39 study heath facilities

Type of equipment	Regional Lab	District lab^a^	Primary Lab	Total
Biochemistry analyser^b^	4	14	13	31
FACS machine	7	6	1	14
Haematology Analyzer^b^	6	6	15	27
HemoCue®	4	2	22	28
Microscopes	2	2	12	16
Pima CD4	0	1	10	11
Refrigerator	2	11	17	30
Others	5	5	21	31

*Lab* Laboratory

^a^Included laboratories located at district and other types of hospitals

^b^Two dispensaries in Iringa urban and Mtwara districts had at least one biochemistry and/or a haematology analyser

## Discussion

The findings of this study showed that whereas majority of the HFs particularly dispensaries were located in rural areas, a larger proportion of laboratories and CTCs were located in urban areas. The study also showed that all hospitals (at regional and district levels) and most of the HCs had laboratories but few dispensaries (36.0%) had laboratories. However, a significantly large number of dispensaries with laboratories were located in urban compared to rural areas. Furthermore, there was low coverage of CTCs especially among areas served by dispensaries (only 15.0% of the dispensaries had CTCs compared to 61.0% of the HCs and 84.6% of the hospitals). This is contrary to the national guidelines which stipulate that all hospitals and health centres should provide HIV diagnostic services for detection of infection status and supportive tests for management and care of PLHA [18, 30]. Furthermore, the low coverage of HIV care and treatment services at low level HFs and in rural areas, which serve the majority of the population [31], suggests that most of the PLHA may have limited access to the critically needed services. However, with laboratories in 13.4% of the dispensaries and most of the health centres (95.2%) in rural areas, there exists a room for scaling up of HIV diagnostic and supportive services in the country.

Although the selected health facilities had CTCs and were expected to be providing HIV diagnostic and related services, it was noted that some hospitals and health centres were not giving the service at the time of the survey. Whereas one of the facilities was a recently established district hospital which was still depending on the regional hospital, the reasons for lack of such services in other facilities contrary to directives of the Ministry of Health [18, 30] could not be established. With the exception of few HFs which were not testing for HIV, all study laboratories were performing screening and routine detection of HIV using rapid diagnostic kits and none of the four regional laboratories was using ELISA as recommended by the National Guidelines [15, 21]. Furthermore, only three laboratories reported that they adopted a new technique in the past three years. Although it was reported that majority of the laboratories were doing internal QC and more than half were taking part in external QA programmes, there was no evidence to support such reports due to lack of documentation. These findings indicate that the laboratories were possibly not adhering to the recommended standards of HIV diagnostic services according to National and International guidelines [26, 32].

Unlike previous studies which showed that most of the laboratories had limited infrastructure [14, 15, 29], this study showed that the facilities have undergone a significant improvement with the exception of working space and waste disposal facilities in some. Further reports have shown that all regional laboratories have undergone major renovations and improvements with the deployment of modern equipment and some (e.g. Tanga Regional Laboratory) are participating in national and international accreditation programmes (I. Semagango, pers. comm.). Availability of key equipment in the study laboratories especially at regional and district level was generally good and harmonization of some of the equipment (e.g. FACS machines) was maintained as recommended by the National Guidelines [15, 21]. The variability in the types of biochemical and haematology analyzers was reported and this has been previously attributed to multiple suppliers and procurement by different donors [15, 21]. Overall, the majority of the equipment (>81) were functional, though about two-thirds had not been serviced in the past three years suggesting that precision and accuracy of the laboratory results could not be guaranteed. With respect to key equipment (biochemical and haematology analyzers), it was also noted that 20–30% were not functional at the time of the visit. This suggests that the problems of after sale service and maintenance which were identified at the initiation of the HIV care and treatment plan have not been resolved [14, 15, 21, 29]. Due to the importance of servicing, maintenance and calibration of equipment on the quality of results they generate, more studies are required and local solutions are needed so that new strategies can be implemented by the Ministry of Health to ensure proper and optimal functioning of equipment.

The study also showed that majority of the HFs were understaffed and facilities located in urban areas had more laboratory personnel compared to those located in rural areas. Although the national guidelines require that low level laboratories should have not less than two laboratory staff [33], this study showed that some laboratories had only one person and two of these had no qualified personnel indicating that availability and accessibility of services in such facilities could be limited. Shortage of health care workers particularly in rural areas with limited infrastructure and social services has been attributed to the low output of qualified staff, low uptake by employment, deployment, retention and higher attrition of skilled staff [34, 35]. Together with limited coverage of services in rural areas, availability of fewer laboratory staff and particularly the technicians further suggest that patients from these areas might have limited access to good quality care and treatment services for HIV/AIDS. Due to the critical shortage of health care workers in Tanzania, a task-shifting strategy has been recommended [36, 37] to avoid disruption of service provision and that can possibly account for involvement of unqualified staff in provision of laboratory services. However, replacement of technical staff with unqualified personnel leads to poor quality of services especially in health facilities where such staff deployed to new departments are not adequately supervised.

### Study limitations

The design and selection of districts and study health facilities are the major limitation which need to be taken into account when interpreting the findings this study. Although the study aimed at recruiting an equal number of hospitals, health centres and dispensaries, majority of the HFs covered were HCs because of availability of fewer hospitals in the study areas and low numbers of dispensaries with both laboratories and CTCs. Thus, generalization of our findings may not be representative for the whole country and might be limited to the districts and regions covered by this study.

## Conclusion

This study showed that diagnostic services for HIV were available in most of the health centres and hospitals while few dispensaries were providing the services. The laboratories had shortage of staff, most of the equipment had not been serviced and fewer HFs participated in quality assurance programmes with possible impacts on the performance of laboratories and quality of services provided by the laboratories. To ensure that laboratories provide good quality services, we recommend strengthening of laboratory systems including human resource, implementation of quality assurance programmes and sustainable preventive maintenance services at all levels in both rural and urban settings.

## Abbreviations

AIDS: Acquired immunodeficiency syndrome
ART: Antiretroviral therapy
CTC: Care and treatment centre
EQA: External quality assurance
GLP: Good laboratory practices
HFs: Health facilities
HIV: Human immunodeficiency virus
MRCC: Medical research coordinating committee
NHLS: National health laboratory system
NIMR: National Institute for Medical Research
PLHA: People living with HIV/AIDS
QA: Quality assurance
QC: Quality control
RPCs: Resource poor countries
THMIS: Tanzania HIV and malaria indicator survey
WHO: World Health Organization
